# Application of massive parallel sequencing to whole genome SNP discovery in the porcine genome

**DOI:** 10.1186/1471-2164-10-374

**Published:** 2009-08-12

**Authors:** Andreia J Amaral, Hendrik-Jan Megens, Hindrik HD Kerstens, Henri CM Heuven, Bert Dibbits, Richard PMA Crooijmans, Johan T den Dunnen, Martien AM Groenen

**Affiliations:** 1 Animal Breeding and Genomics Centre, Wageningen University, PO Box 388, Wageningen, 6700 AH, The Netherlands; 2 Utrecht University, 3508TD, Utrecht, The Netherlands; 3 Leiden Genome Technology Center, Leiden University Medical Center, Leiden, The Netherlands

## Abstract

**Background:**

Although the Illumina 1 G Genome Analyzer generates billions of base pairs of sequence data, challenges arise in sequence selection due to the varying sequence quality. Therefore, in the framework of the International Porcine SNP Chip Consortium, this pilot study aimed to evaluate the impact of the quality level of the sequenced bases on mapping quality and identification of true SNPs on a large scale.

**Results:**

DNA pooled from five animals from a commercial boar line was digested with *Dra*I; 150–250-bp fragments were isolated and end-sequenced using the Illumina 1 G Genome Analyzer, yielding 70,348,064 sequences 36-bp long. Rules were developed to select sequences, which were then aligned to unique positions in a reference genome. Sequences were selected based on quality, and three thresholds of sequence quality (SQ) were compared. The highest threshold of SQ allowed identification of a larger number of SNPs (17,489), distributed widely across the pig genome. In total, 3,142 SNPs were validated with a success rate of 96%. The correlation between estimated minor allele frequency (MAF) and genotyped MAF was moderate, and SNPs were highly polymorphic in other pig breeds. Lowering the SQ threshold and maintaining the same criteria for SNP identification resulted in the discovery of fewer SNPs (16,768), of which 259 were not identified using higher SQ levels. Validation of SNPs found exclusively in the lower SQ threshold had a success rate of 94% and a low correlation between estimated MAF and genotyped MAF. Base change analysis suggested that the rate of transitions in the pig genome is likely to be similar to that observed in humans. Chromosome X showed reduced nucleotide diversity relative to autosomes, as observed for other species.

**Conclusion:**

Large numbers of SNPs can be identified reliably by creating strict rules for sequence selection, which simultaneously decreases sequence ambiguity. Selection of sequences using a higher SQ threshold leads to more reliable identification of SNPs. Lower SQ thresholds can be used to guarantee sufficient sequence coverage, resulting in high success rate but less reliable MAF estimation. Nucleotide diversity varies between porcine chromosomes, with the X chromosome showing less variation as observed in other species.

## Background

The Sanger DNA sequencing technique has been and still is the method of choice for *de novo *sequencing of complete genomes [1,2]. However, whole genome sequencing using the Sanger method is relatively expensive, labor intensive, and time consuming.

Several methods for ultra high-throughput DNA sequencing that reduce the cost and labor demands of Sanger sequencing are currently available [3,4]. The Illumina 1 G Genome Analyzer (ILLUMINA, San Diego, CA, USA) uses a sequencing by synthesis method, during which millions of DNA fragments are sequenced in parallel (massive parallel sequencing). With this method, costly and often problematic procedures, such as cloning are eliminated. Another advantage is that accuracy is independent of sequence context because a discrete signal is generated per each base. Thus, this method is very accurate in cases of homopolymeric sequences and generates quality values that are analogous to Phred scores [5]. Sequence lengths generally range from 25–50 bp (short sequences), which is sufficient for unique alignment to a reference genome [6]. Because millions of fragments are sequenced in parallel, a fragment can be sequenced even if it exists in low abundance in the sample, thereby increasing sequencing depth and enabling identification of single nucleotide polymorphisms (SNPs) with high accuracy [7-9].

Sequencing of reduced representation libraries (RRLs), which are reproducible subsets of the genome, allows cost-effective genome-wide SNP discovery with accurate estimations of minor allele frequencies (MAF) [10]. Because the cost of large-scale sequencing of RRLs is still prohibitive for individual samples, DNA samples can be pooled to further reduce sequencing costs and simultaneously infer the frequencies of two SNP alleles with high accuracy [11]. Previous studies have shown that large-scale SNP discovery can be accurate using massive parallel sequences of RRLs prepared from pooled DNA [8,12].

Despite the efficiency of massive parallel sequencing for providing large amounts of sequencing data, a sequence selection stage is still required. Previous studies have applied various rules for sequence selection: sequences must start with a restriction motif [8], sequences must be aligned to a unique location in the genome [9], and sequences must have a minimum average sequence quality (SQ) score of 20 [8,9]. An effective sequence selection stage can decrease noise in the data that can compromise alignment and SNP identification. Therefore, the effect of different levels of SQ in identifying SNPs needs to be evaluated.

With increasing attention being paid to genomic selection by animal breeders, there is a need for high-density SNP maps of the genomes of farm animals. Experimental evidence has shown that linkage disequilibrium extends from 0.1 to 2 cM in European commercial pig breeds [13]; thus, an SNP assay should contain a minimum of 30 k informative SNPs. To achieve this goal, we designed a cost-effective strategy for large scale identification of SNPs in the porcine genome that could be applied to other species.

In this study, an RRL generated from a DNA pool of a boar line was sequenced using the Illumina 1 G Genome Analyzer. The two main goals of our study were (a) to develop rules for decreasing sequence ambiguity (sequence alignment to several locations in the genome), which would decrease noise and increase efficiency in sequence alignment and SNP identification, and (b) to evaluate the effects of different SQ filtering strategies for cost-effective, large-scale identification of SNPs.

## Results and discussion

### Sequencing and filtering the RRL

An RRL was produced from a DNA pool of five boars from a crossbred (Large White *vs*. Pietrain) commercial boar line (PW), using the restriction enzyme *Dra*I, which recognizes pattern "TTTAAA" and generates blunt-ended fragments starting with AAA. Fragments 150–250-bp long were selected and end-sequenced using the Illumina 1 G Genome Analyzer. Short sequences will align to a unique genomic location (target), creating groups (clusters) with a number of sequences (target depth) sufficient for SNP identification. The *in silico *digest of pre-EnsEMBL *Sus scrofa *build 7 [14] indicated an expected sequence coverage of ~1% of the reference genome, that is, 11,089,914 bp uniquely aligned to the porcine genome.

In total, 70,348,064 sequences were generated during three different runs. In addition to sequence information, this second generation platform generates quality scores that are analogous to Phred scores (which assign a probability to the four possible nucleotides for each base in the sequence) [15]. Levels of base quality varied between runs and along the sequence length, and decreased considerably at the 3'end [see Additional file 1]. This variation in base quality along the sequence has been reported in previous studies using short sequencing [9]. Base quality for the first three bases is poor, but increases before decreasing again at the end of the 31-bp sequence. Poor base quality at the first three bases is due to the properties of the algorithm implemented in the Illumina base caller BUSTARD^® ^(ILLUMINA, San Diego, CA, USA). The quality score of a base is calculated by comparing the fluorescence signals of the previous and following bases. The algorithm does not expect a repeated motif in the beginning of a sequence (AAA) and therefore estimates poor quality scores. The severe decrease in base quality at the 3' end of the sequence indicates the existence of a higher level of sequencing errors at the 3' end. The proportion of unique sequences (sequences occurring only once) ranged from 35% when considering a sequence length of 29 bp to 55% when considering a sequence length of 35 bp, which again indicates an increase in sequencing errors with an increase in sequence length. Therefore, sequences were trimmed at 33 bp and filtering rules were applied.

We applied a number of rules to select sequences for further analysis: (a) screening for properties of the RRL (i.e., discarding sequences without the restriction motif "AAA" in the 5' end); (b) filtering for sequence ambiguity, and (c) filtering for SQ. Filtering for sequence ambiguity was performed by removing sequences with homopolymers and removing sequences with a high rate of re-sampling. Sequences with a re-sampling rate above an expected level were discarded as potentially paralogous sequences. Because pre-EnsEMBL *Sus scrofa *build 7 [14] comprises approximately 70% of the porcine genome, unique alignment of reads does not guarantee that there is no other similar (duplicated) sequence in the remainder of the genome. Therefore, potential paralogous sequences should be eliminated from the data set to avoid identification of false-positive SNPs. This was done by estimating the ratio between the total number of fragments obtained after filtering for the restriction motif, unknown bases, homopolymers, and the numbers of fragments generated from an *in silico* digest. This ratio, the estimated average level of sequence re-sampling, was estimated at 35×, and sequences with a frequency approximately 3-fold greater (>100×) were removed.

The Illumina 1 G Genome Analyzer^® ^produces quality scores analogous to PHRED scores. SQ has been defined in previous studies as the average of the quality scores of all bases in a sequence, and the threshold has been set to a minimum of SQ = 20 [8,9], which implies that on average, 1 in 100 bases is wrongly identified. Applying this strict filtering rule left sufficient target coverage for SNP identification. In this study, we aimed to evaluate the impact of different thresholds of SQ on the identification of true SNPs. SQ was also evaluated by calculating the average of the base quality scores for all the bases of a given sequence. Three data sets with different SQ levels (12, 15, and 20) were generated and compared for SNP identification. These three different data sets are hereafter referred to as Data 12 for a quality level of 12, Data 15 for a quality level of 15, and Data 20 for a quality level of 20. The total number of sequences that remained after applying all of the filtering rules and that were used for alignment with the reference genome for Data 12, Data15, and Data 20 are shown in Table 1.

**Table 1:** Sequence production and filtering for the three strategies used to identify SNPs.

	**Data 12**	**Data 15**	**Data 20**
Total sequences after filtering	45,498,558	41,610,684	34,061,918
Total number of SNPs	16,768	17,047	17,489
Mapping quality^a^	61.76 (0.027)	61.78 (0.027)	62.02 (0.0237)
Consensus quality^a^	59.04 (0.257)	60.23 (0.259)	63.30 (0.263)
Target coverage^a^	29.37 (0.164)	29.19 (0.163)	28.60 (0.155)
MAF^a, b^	0.36 (0.0007)	0.36 (0.0007)	0.36 (0.0007)

^a ^Mean (s.e.)

^b ^MAF, minor allele frequency.

### Comparison of strategies for SNP identification

**Figure 1:**
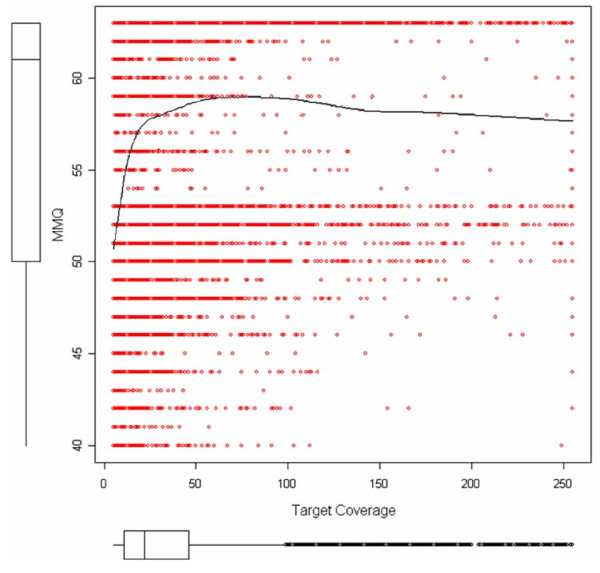
**Maximum mapping quality (MMQ) (mapping quality of the best mapped sequence of a cluster) on an SNP position versus target coverage**. Box plots show the data distribution for each parameter. Red dots show MMQ values for the best mapped sequence on an SNP position versus target coverage. The black solid line shows the smooth-fit line.

Sequence mapping was performed using an algorithm that calculates the probability that a sequence maps to a specific target in the genome [16]. Filtered sequences of Data12, Data15, and Data 20 were mapped to pre-EnsEMBL *Sus scrofa *build 7 [14]. Mapping quality (which is the probability with which sequences were aligned to a unique location in the genome) was very similar between the three strategies (approximately 60; Table 1). This value indicates an error in the mapping procedure of approximately 1/6000 sequences [16]. After mapping, consensus sequences were generated and SNPs were extracted, creating a large set of potential SNPs. At this stage, the algorithm identified 1,703,360 potential SNPs in Data 12, 1,541,991 potential SNPs in Data 15, and 1,193,814 potential SNPs in Data 20. Four filters were then applied to decrease the rate of false-positive SNPs: 1) SNPs were only accepted if they were identified in targets to which only non-ambiguous sequences were assigned; 2) the maximum mapping quality (mapping quality of the best mapped sequence of a cluster) of the target was larger than or equal to 40; 3) the minimum mapping quality (mapping quality of the sequence with the lowest mapping quality) of a target should be 10 or greater, and 4) the consensus quality (CQ), which measures the probability of the existence of a polymorphism, was 10 or greater (90% of the identified SNP are true positives). Figure 1 shows the relationship between target coverage and mapping quality. The smooth line shows a decrease after target coverage exceeds 100 sequences. This indicates that clusters with a level of target coverage above the expected number calculated from the *in silico *analysis have a lower mapping quality and are less reliable for SNP identification. Additional filters were used to further decrease the rate of false-positive SNPs: 1) occurrence of the minor allele in a minimum of three sequences (to increase the accuracy of detecting SNPs with high MAF), and 2) a maximum target coverage of 100 reads. Again, the restriction of maximum target coverage aims to decrease the rate of false-positive SNPs identified in potential paralogous regions that align to each other because the available assembly only comprises around 70% of the total pig genome. The results allowed us to identify a larger number of SNPs in Data 20 (Table 1) with a higher level of CQ, lower target coverage, and similar MAF values as compared to Data 12 and Data 15.

**Figure 2:**
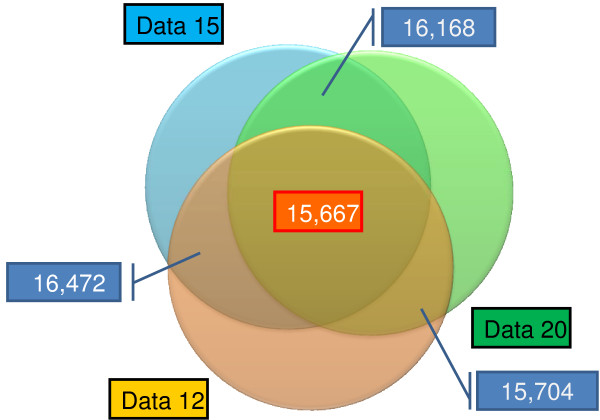
**Venn diagram showing the number of identical SNPs between the analyzed data sets with different levels of sequence quality**.

Although a larger set of sequences was used in Data 12, resulting in a higher number of potential SNPs, the actual number of true SNPs was lower due to the removal of more false positives in the final round of filtering. This indicates that a large number of sequences from this data set were mapped ambiguously, introducing noise into the analysis, and shows that the application of filters for SNP selection is crucial for decreasing the rate of false positives. Because the DNA pool contained 10 genomes and the threshold for the minor allele count was three sequences, the observed MAFs are greater than 0.1 for all strategies analyzed [see Additional file 1] and quite adequate for the use of these SNPs in whole genome association and genomic selection studies. For a higher level of SQ (Data 20), more SNPs with MAFs between 0.1 and 0.2 were identified. This indicates that in SNP discovery studies aimed at identifying rare SNPs, greater sequence depth and higher levels of SQ are advisable. A large number of the SNPs identified using Data 12 were also identified using Data 15 and Data 20 (Figure 2) as a result of the CQ threshold used in the analysis (90% correct SNP calling rate). Our results indicate that in cases of lower target coverage, lowering the SQ threshold may increase the SNP discovery rate while keeping the rate of false-positive SNPs low.

**Figure 3:**
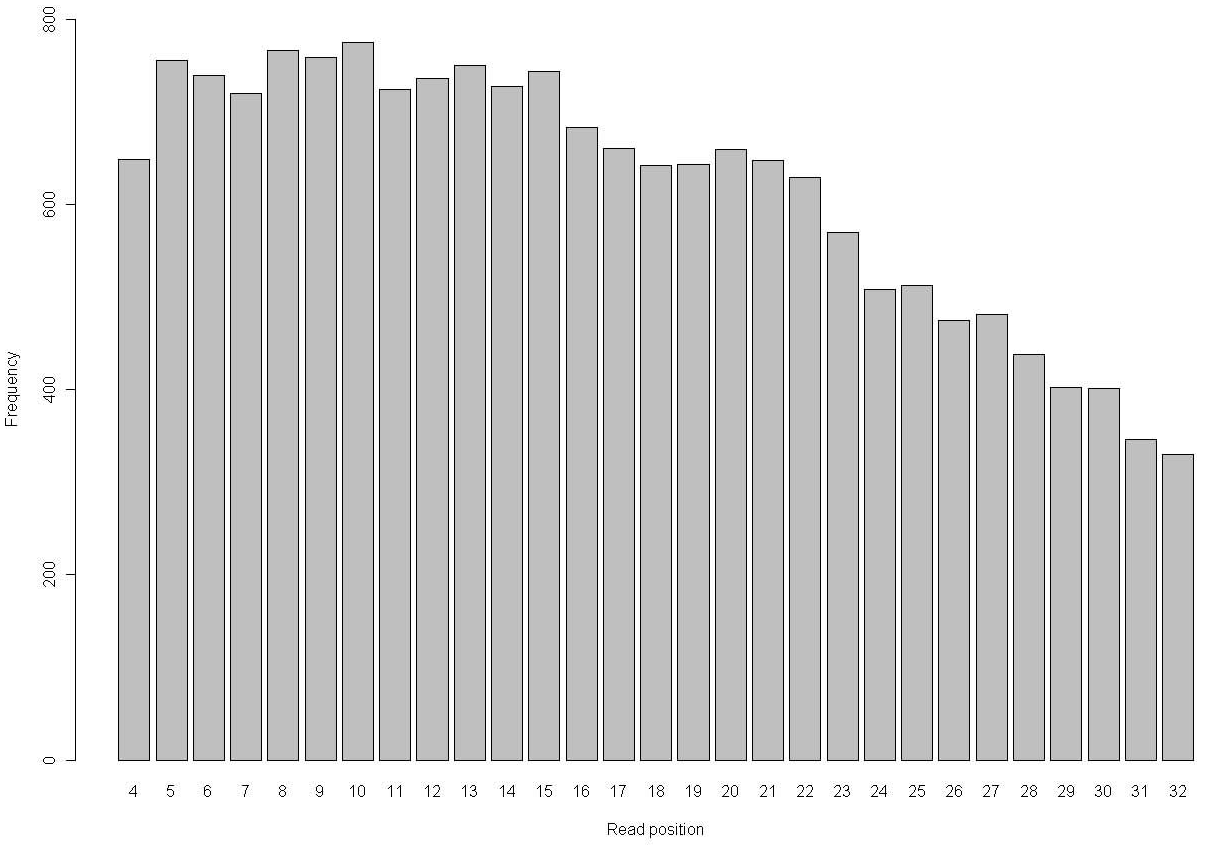
**Number of identified SNPs per position in a short read for Data 20**.

The decrease in quality at the 3' end of the sequences affected the number of SNPs found per position in the sequence reads. Figure 3 shows that the number of SNPs identified decreased from the 5' to the 3' end, indicating that with the strict rules for SNP selection used in our study, base errors in the 3' end were not incorrectly identified as SNPs.

### RRL sequence coverage along the pig genome

The reference genome was digested *in silico *and the predicted coverage compared to that of the aligned consensus sequences is shown in Table 2. The consensus sequences aligned evenly to all chromosomes, indicating that the obtained RRL represents a good random sample of the genome. Table 2 also shows that for all chromosomes, the total sequence length that uniquely aligned to the reference genome was somewhat greater than the value expected from the *in silico *digest, most obviously for SSC7. This could have resulted from inadequate resolution of DNA fragments during electrophoresis, leading to selection of fragments larger than 150–250 bp and resulting in 13,376,663 bps of aligned sequences, significantly more than the 11,089,914 bps expected from the *in silico *digest of the genome assembly [14].

**Table 2:** Summary of *in silico *digest of reference genome and analysis of consensus sequences.

**Chr**	**Total sequence length (bp)** ***in silico***	**Total observed sequence length (bp)**	**Total observed sequence length (bp) per 1-Mb window**		**Nucleotide diversity**		**GC content (%)**	
			
			**Mean**	**s.e**.	**Mean**	**s.e**.	**Mean**	**s.e**.
1	1,942,512	2,167,060	8,738.15	204.29	0.0013	0.00007	31.72	0.11
2	564,894	704,052	8,912.05	430.80	0.0017	0.00015	31.82	0.21
3	396,990	510,151	7,971.11	337.50	0.0013	0.00011	32.60	0.22
4	931,194	988,690	7,724.14	286.62	0.0012	0.00009	32.26	0.16
5	541,200	589,399	7,805.12	417.18	0.0019	0.00012	32.29	0.24
6	263,538	354,332	7,874.04	508.06	0.0015	0.00013	32.65	0.24
7	263,538	831,673	6,253.18	283.95	0.0015	0.00010	32.62	0.17
8	590,700	732,338	10,930.42	338.24	0.0011	0.00013	31.36	0.22
9	582,384	763,287	9,541.09	406.33	0.0014	0.00014	32.06	0.18
10	292,842	367,327	8,959.20	380.01	0.0021	0.00019	32.44	0.21
11	527,274	584,831	9,137.98	370.76	0.0015	0.00013	31.85	0.30
12	135,894	174,581	6,020.03	462.41	0.0018	0.00015	33.71	0.36
13	924,396	1,177,791	9,897.40	293.47	0.0014	0.00009	31.64	0.14
14	874,500	952,048	6,476.52	265.24	0.0014	0.00008	32.83	0.17
15	822,822	974,572	10,185.12	326.63	0.0010	0.00009	31.47	0.14
16	402,270	500,390	10,007.80	481.24	0.0016	0.00013	31.97	0.26
17	280,434	303,111	5,511.11	367.98	0.0015	0.00011	33.22	0.24
18	256,806	314,098	9,518.12	593.62	0.0007	0.00010	32,45	0.29
X	495,726	386,932	5,300.44	181.11	0.0005	0.00009	31.77	0.20

### Sequence polymorphism in the pig genome

**Figure 4:**
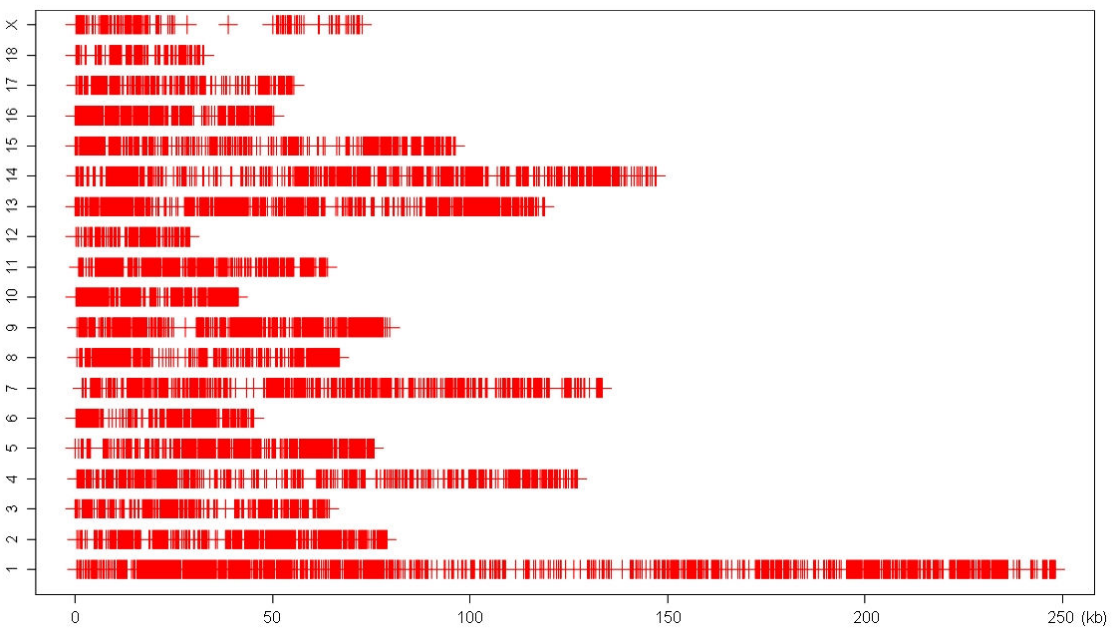
**SNP map of each chromosome based on Data 20**. The colored vertical lines represent the location of each SNP.

Figure 4 shows the SNP map obtained for the SNP identification strategy with an SQ level of 20. A total of 17,489 SNPs were identified and, as expected, more SNPs were found on chromosomes for which more sequence was available in the build of the reference genome [14]. Therefore, chromosomes SSC1, SSC4, and SSC14 contain the largest number of SNPs identified. When analyzing the nature of the base changes, we found that transitions were more frequent than transversions and comprised 67.15% of the identified SNPs. This transition-to-transversion ratio is similar to the 2:1 ratio in observed in the human genome [17]. As well, this frequency agrees with that reported in an earlier porcine SNP discovery study [18].

**Figure 5:**
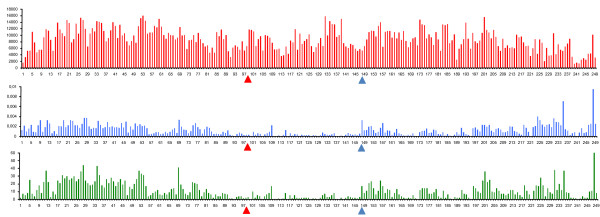
**Sequence coverage, nucleotide diversity, and SNP occurrence along chromosome 1**. Each bar represents a window of 1 Mb. Red bars show the length of the aligned consensus sequence, blue bars show the estimated level of nucleotide diversity, and green bars show the number of SNPs found in each window. The red triangle designates the position of the centromere. The blue triangle designates a position where nucleotide diversity is high where coverage is low.

Nucleotide diversity [19] across all chromosomes was evaluated in 1-Mb windows based on the pre-EnsEMBL *Sus scrofa *build 7 [14]. This analysis showed that SSC 5, SSC10, and SSC12 have relatively greater nucleotide diversity, whereas SSC18 and SSCX have relatively lower nucleotide diversity (Table 2). Figure 5 illustrates the variation in nucleotide diversity and the length of sequence coverage for SSC1. Regions towards the telomeres have greater levels of nucleotide diversity and regions close to the centromere have the lowest levels of nucleotide diversity. The results for window 149, shown in Figure 5, also indicate that in windows of lower sequence coverage, nucleotide diversity may be overestimated. For some chromosomes (SSC4, SSC8, and SSC15), a correlation was observed between the level of GC content and nucleotide diversity, suggesting a relationship between GC content and polymorphism patterns for specific chromosomal regions. Previous studies have shown a relationship between GC content and polymorphism patterns in humans. Such GC-rich regions have been identified as regions of gene conversion and recombination hot spots [20]. Our results suggest that such relationships exist in many porcine chromosomes.

Although this study covered only ~1% of the porcine genome, using an RRL allowed estimation of genome-wide nucleotide diversity. Variation in nucleotide diversity and length of sequence coverage for the remaining chromosomes are shown in [see Additional file 1]. The pattern of variation in nucleotide diversity along chromosomes varies between chromosomes; SSC4, SSC8, SSC9, SSC10, SSC13, SSC15, and SSCX have higher levels of nucleotide diversity towards the telomeric regions and lower levels of nucleotide diversity in the centromeric region. On SSCX, large areas flanking the centromere were devoid of nucleotide diversity. Reduced variability in the X chromosome relative to the autosomes has been described for other species, including humans [21,22], *Drosophila *[23], and mice [24], and is explained mainly by the fact that this chromosome has a lower mutation rate and a smaller effective population size [25].

### SNP genotyping and validation

A SNP chip assay was conducted to validate a sample of 3,230 SNPs in the original SNP discovery panel. Of the 3,230 SNPs, 3% failed as a result of the assay design. The validation assay included 68 assayable SNPs exclusively found in Data 12, 147 assayable SNPs exclusively shared between Data 12 and Data 15, 48 assayable SNPs exclusively found in Data 20, and 2,879 assayable SNPs shared between Data 12, Data 15, and Data 20. The correlation between the estimated MAF (calculated from the analysis of short sequences) and the genotyped MAF in the animals used in the discovery panel was calculated for the 2,879 SNPs shared between Data 12, Data 15, and Data 20. The observed correlation of 0.32 was somewhat lower than that reported by Wiedmann [12]. In order to investigate this result, MAF obtained from short sequence data and from genotyping were simulated and correlations were calculated. Results from simulations showed that the correlation between MAF estimated from short sequences and MAF estimated from genotype data can range from 0.1 to 0.5. Of the total number of SNPs tested, 4% appeared to be monomorphic and 85% had an MAF above 0.2, showing that our strategy yielded a high proportion of informative SNPs useful for whole genome association assays. The fact that the correlation between estimated and observed MAFs was not high could restrict the type of measures used to evaluate genomic variation in population genomics studies using short sequences. For example, estimation of pairwise nucleotide diversity (π) requires accurate estimates of MAF, and a correlation of 0.3 shows that the π estimated from short sequences can be biased.

Table 3 shows that assayed SNPs are also informative for other breeds. Large White and Pietrain, which are the breeds used in the cross of the animals analyzed in the discovery panel, show an average MAF closer to the average MAF of the discovery panel. Duroc has one of the highest rates of monomorphic SNPs and the lowest average MAF. This is in agreement with other diversity studies [26] showing that Duroc is genetically more distant from Large White and Pietrain. The breed with highest rate of monomorphic SNPs, Hampshire, has also been reported by previous studies to present high level of genetic differentiation in comparison with Large White [26]. The average MAF is higher for PW (the commercial boar line used in the discovery panel), probably due to the criteria used for SNP selection, which demanded observation of the minor allele in a minimum of three sequences. For the 68 SNPs identified exclusively in Data 12 and in Data 15, 7% were monomorphic and the correlation between the estimated MAF and the genotyped MAF was 0.08 in the animals used for the discovery panel. This indicates that highly accurate SNP identification is possible even when using a lower SQ threshold for SNP identification, although the cost is less precise estimation of the MAF.

**Table 3:** Percentage of monomorphic SNPs and average minor allele frequencies (MAF) by breed for 3,142 SNPs.

		**Data 12** **# SNPs = 68**		**Data 12 & Data 15** **# SNPs = 147**		**Data 20** **# SNPs = 48**		All* # SNPs = 2,879	
		
**Breed**	**# Genotyped Animals**	**Monomorphic** **(%)**	**Average** **MAF**	**Monomorphic** **(%)**	**Average** **MAF**	**Monomorphic** **(%)**	**Average** **MAF**	**Monomorphic** **(%)**	**Average** **MAF**
Duroc	82	34	0.13	28	0.16	17	0.17	29	0.13
Large White	136	13	0.23	7	0.24	8	0.28	5	0.24
Landrace	80	12	0.22	12	0.20	17	0.20	11	0.21
Pietrain	90	16	0.19	12	0.22	10	0.26	12	0.22
Berkshire	67	32	0.14	31	0.14	21	0.18	28	0.14
Hampshire	59	28	0.15	28	0.16	23	0.17	27	0.16
Wild boar	20	34	0.17	25	0.19	17	0.20	23	0.18
PW	6	13	0.27	6	0.33	6	0.37	4	0.33

*SNPs identified in Data 12, Data 15, and Data 20.

## Conclusion

We presented a strategy for using short sequences derived from second generation sequencing technology to efficiently identify large numbers of SNPs with MAF estimates at a low false discovery rate. These results show that by lowering the SQ it is possible to identify SNPs while still keeping the false discovery rate low, although the cost is a lower correlation between the estimated and true MAFs. Finally, our data show that nucleotide diversity is quite variable among porcine chromosomes and is particularly low on SSCX.

## Methods

### Library construction and sequencing

DNA was extracted from five individual boars produced from a cross between Landrace and Pietrain. Extracted DNA was pooled (60 ng) and digested with *Dra*I (100 units; New England Biolabs, Ipswich, MA, USA) at 37°C for 16 hours. The fragments were separated by 1.2% agarose gel electrophoresis (2 hours; 60 volts), and 150–250 bp-fragments were eluted (yielding 1069 ng), end-repaired, and ligated to Illumina's oligonucleotide adapters. Fragments were end-sequenced using the Illumina 1 G Genome Analyzer.

The Illumina 1 G Genome Analyzer generates image information that is translated by BUSTARD^® ^into sequences and base quality scores similar to Phred [15]. Using PERL scripts prepared by the authors, quality scores were converted into PHRED scores.

### Sequence mapping and SNP identification

Filtering rules were applied to select sequences that were mapped to the pig genome [14] using MAQ 0.6.6 [16]. The algorithm implemented in MAQ calculates a mapping quality for each sequence that measures the probability that a sequence belongs to a specific target [16]. Mapping was performed allowing two mismatches and a mutation rate of 0.001. To generate consensus sequences, the algorithm implemented in MAQ estimated CQ, which is the value at which the probability of each genotype is maximized [16]. Consensus sequences were generated allowing a maximum of four mismatches since the expected SNP frequency in pigs is 1/336 bp [27]; therefore, the percentage of clusters with more than one SNP should be low.

### Nucleotide diversity analysis

The total aligned consensus sequence was obtained using option *cns.view *of MAQ [16]. Full output was filtered using the same rules applied to SNP identification. A PERL script was developed by the authors to calculate mapping density, nucleotide diversity [19], and GC content over 1-Mb windows.

### Validation

A total of 3,142 SNPs located in *Sus scrofa *build 7 [14] were validated using the Illumina Infinium^® ^Genotyping assay on an Illumina^® ^BeadStation.

Oligonucleotides were designed, synthesized, and assembled into oligo-pooled assays by Illumina Inc.

Individual DNA samples from the animals used for the SNP identification panel (PW) were genotyped, plus one more animal from PW, 20 samples of Wild Boar, 136 samples of Large White, 80 samples of Landrace, 82 samples of Duroc, 90 samples of Pietrain, 67 samples of Berkshire, and 39 samples of Hampshire.

## Authors' contributions

AJA designed and developed the PERL pipeline for sequence filtering, performed all bioinformatics and statistical analyses, and wrote the manuscript. H-JM was involved in discussions about all of the analyses performed, developed the PERL script to perform nucleotide diversity analysis, GC content, mapping density, and *in silico *digests. HHDK developed a PERL pipeline to parallelize sequence mapping analysis and to extract information for SNP statistical analysis. HCMH was involved in discussions about all of the analyses performed. BD and RPMA collected and prepared the samples for sequencing. MAMG coordinated and supervised the project. JD provided facilities for DNA sequencing. MAMG, H-JM, and HCMH assisted in manuscript preparation. All authors read and approved the final manuscript.

## Additional files and information

The sequences and nucleotide variations have been submitted to GenBank dbSNP database with accession numbers ranging from 120015817 to 120032847.

## Supplementary Material

Additional file 1 **Supplemental Figures 1–3**. Supplemental Figures 1, 2, and 3 cited in manuscript. Click here for file
