# Supplementary material for: Application of massive parallel sequencing to whole genome SNP discovery in the porcine genome
Source: BMC Genomics. 2009 Aug 12;10:374. doi: 10.1186/1471-2164-10-374 (PMC2739861; doi:10.1186/1471-2164-10-374)
Supplement: Additional file 1 — Supplemental Figures 1–3. Supplemental Figures 1, 2, and 3 cited in manuscript. [file 1471-2164-10-374-S1.pdf]

Supplemental Figure 1 - Average quality score per position on the sequence. Results are shown for several runs: Run 1 (red bars, 463,012 sequences), Run 2 (green bars, 14,827,185 sequences) and Run 3 (blue bars, 55,057,867 sequences). Sequences were trimmed at 35 bp.

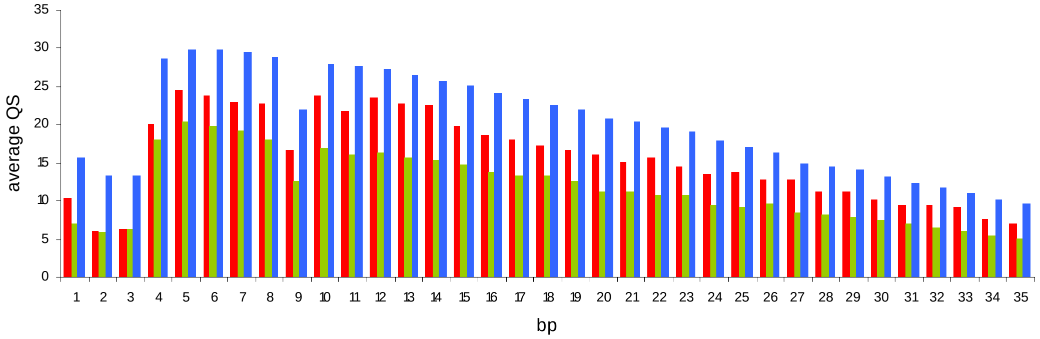

Supplemental Figure 2- Distribution of minor allele frequency for data sets: Data12, Data 15 and Data 20.

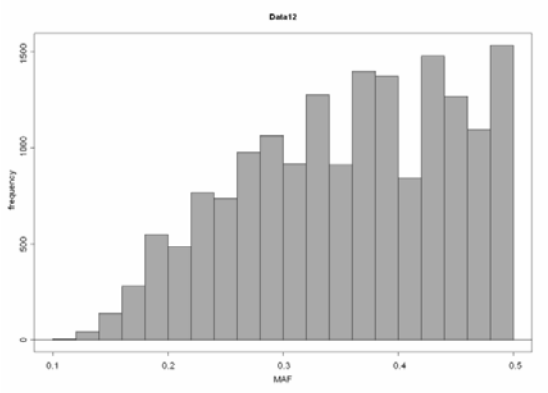

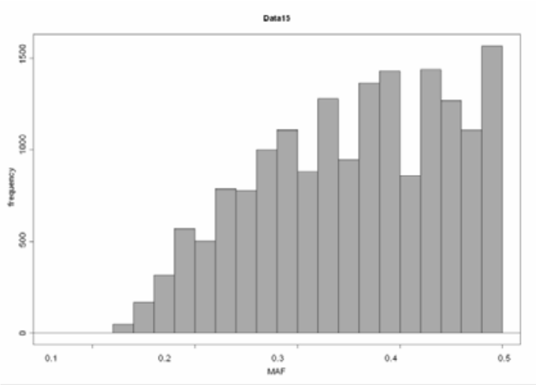

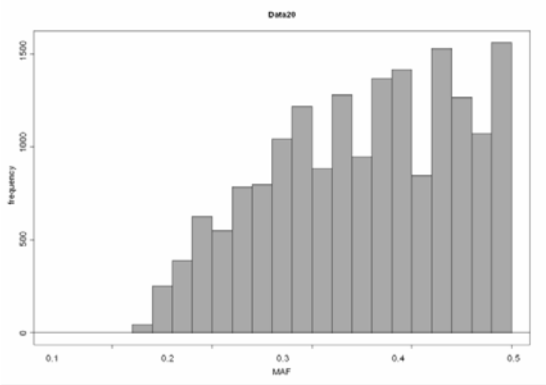

Supplemental Figure 3 - Nucleotide diversity along remaining porcine chromosomes. Each bar represents a window of 1Mb over the chromosome. Red bars indicate chromosome window containing the centromere.

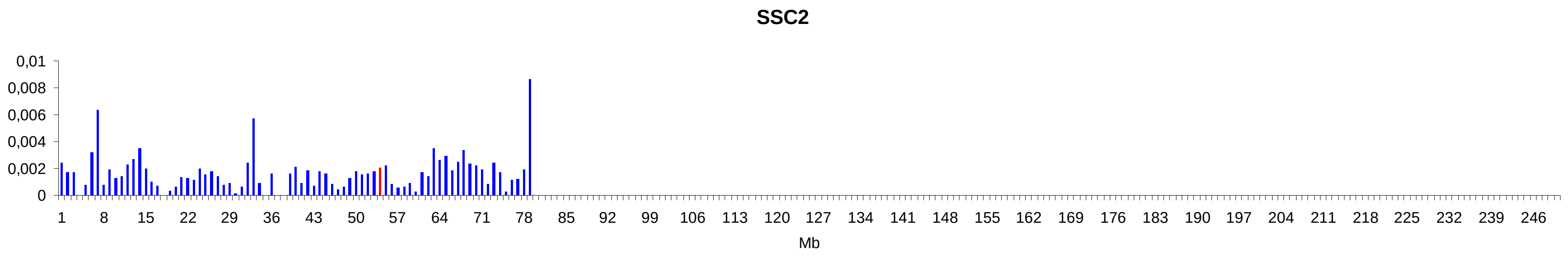

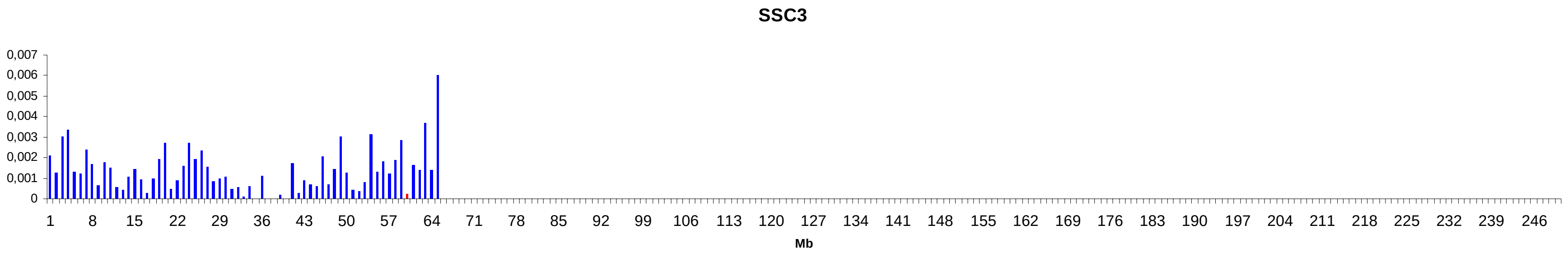

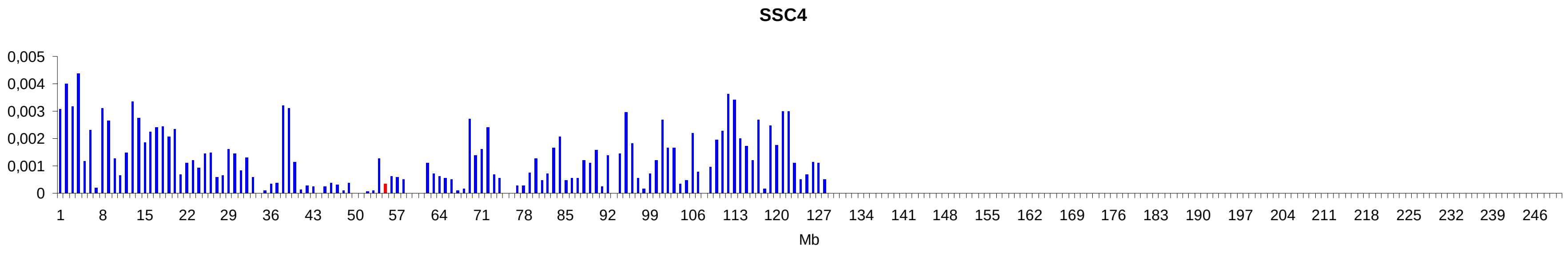
SSC4
0,005
0,004
0,003
0,002
0,001
0
1 8 15 22 29 36 43 50 57 64 71 78 85 92 99 106 113 120 127 134 141 148 155 162 169 176 183 190 197 204 211 218 225 232 239 246
Mb

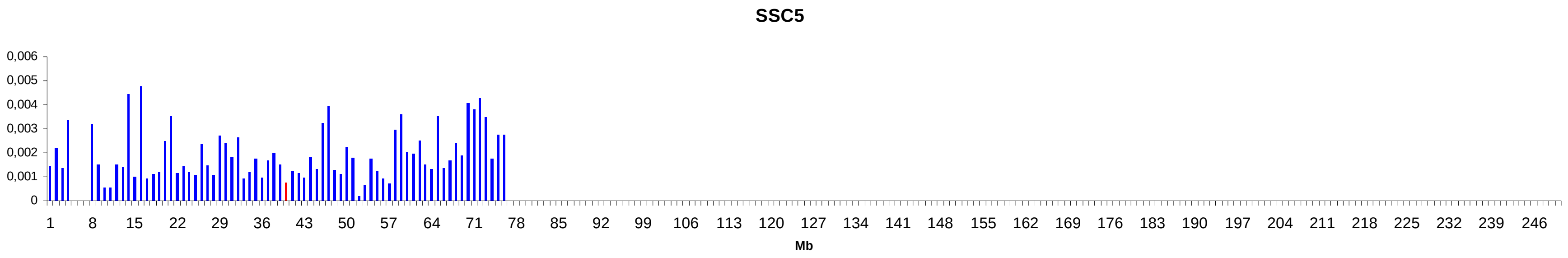
SSC5
0,006
0,005
0,004
0,003
0,002
0,001
0
1 8 15 22 29 36 43 50 57 64 71 78 85 92 99 106 113 120 127 134 141 148 155 162 169 176 183 190 197 204 211 218 225 232 239 246
Mb

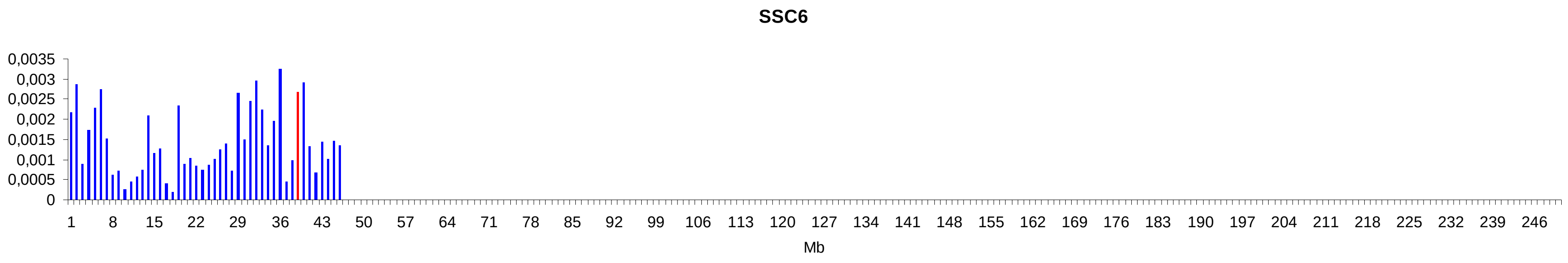
SSC6
0,0035
0,003
0,0025
0,002
0,0015
0,001
0,0005
0
1 8 15 22 29 36 43 50 57 64 71 78 85 92 99 106 113 120 127 134 141 148 155 162 169 176 183 190 197 204 211 218 225 232 239 246
Mb

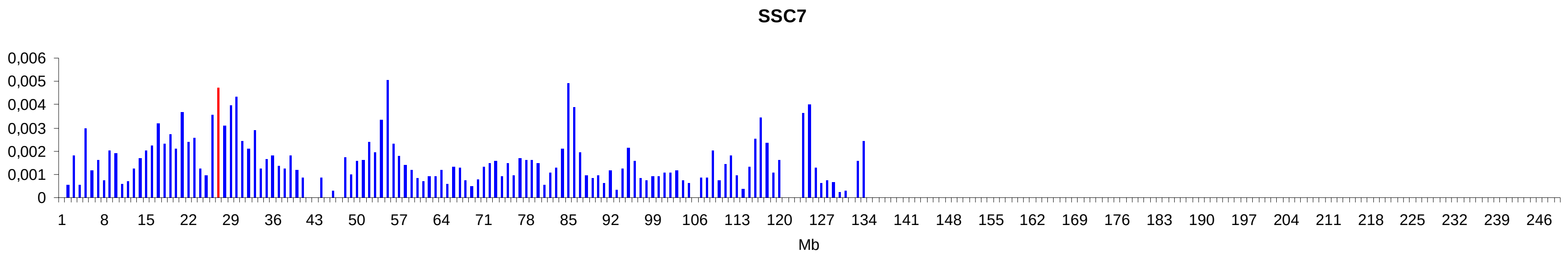
SSC7
0,006
0,005
0,004
0,003
0,002
0,001
0
1 8 15 22 29 36 43 50 57 64 71 78 85 92 99 106 113 120 127 134 141 148 155 162 169 176 183 190 197 204 211 218 225 232 239 246
Mb

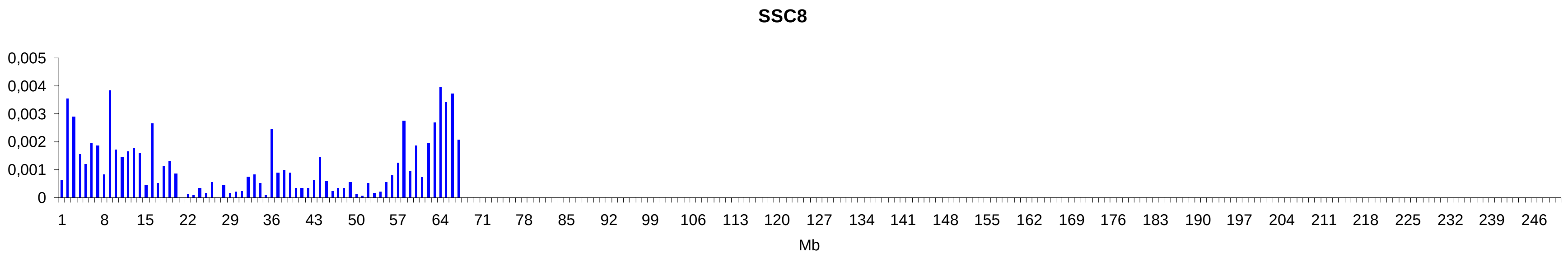
SSC8
0,005
0,004
0,003
0,002
0,001
0
1 8 15 22 29 36 43 50 57 64 71 78 85 92 99 106 113 120 127 134 141 148 155 162 169 176 183 190 197 204 211 218 225 232 239 246
Mb

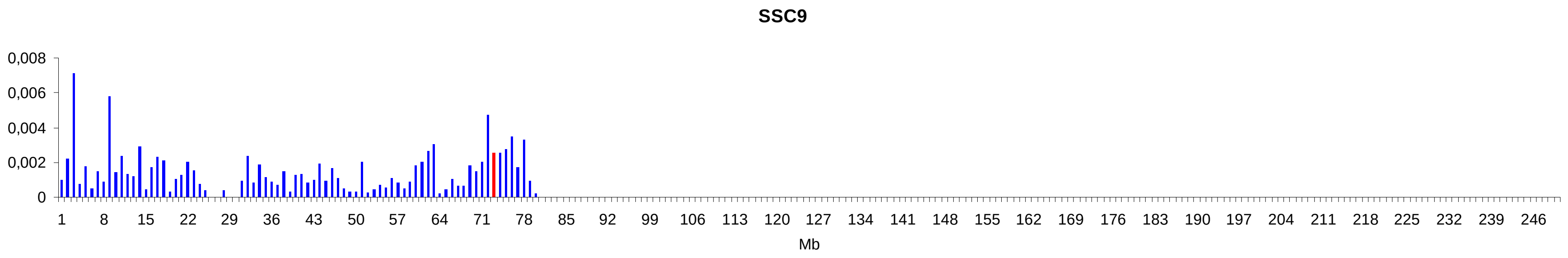
SSC9
0,008
0,006
0,004
0,002
0
1 8 15 22 29 36 43 50 57 64 71 78 85 92 99 106 113 120 127 134 141 148 155 162 169 176 183 190 197 204 211 218 225 232 239 246
Mb

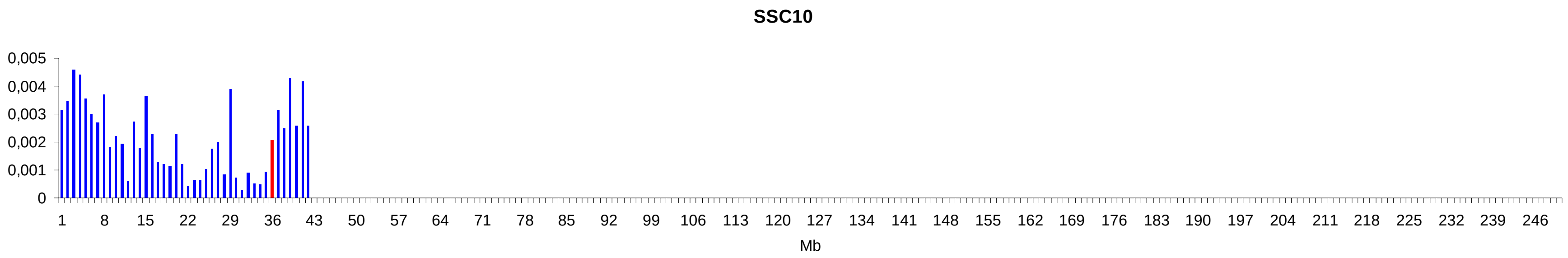
SSC10
0,005
0,004
0,003
0,002
0,001
0
1 8 15 22 29 36 43 50 57 64 71 78 85 92 99 106 113 120 127 134 141 148 155 162 169 176 183 190 197 204 211 218 225 232 239 246
Mb

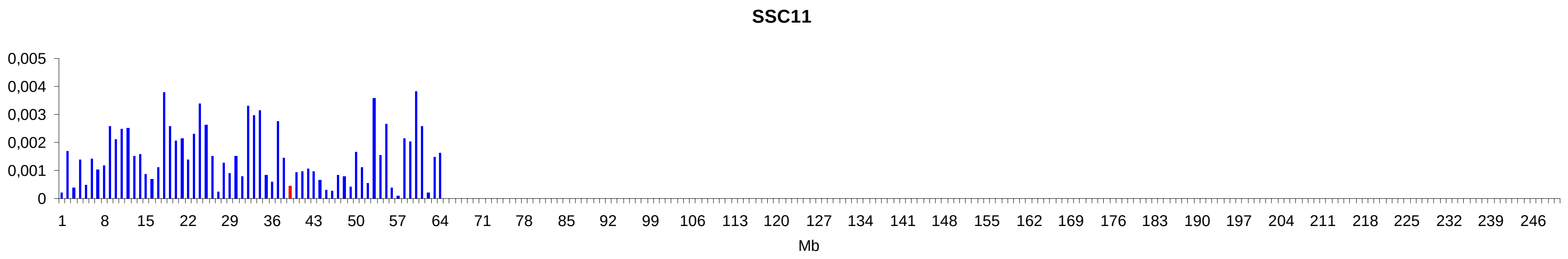
SSC11
0,005
0,004
0,003
0,002
0,001
0
1 8 15 22 29 36 43 50 57 64 71 78 85 92 99 106 113 120 127 134 141 148 155 162 169 176 183 190 197 204 211 218 225 232 239 246
Mb

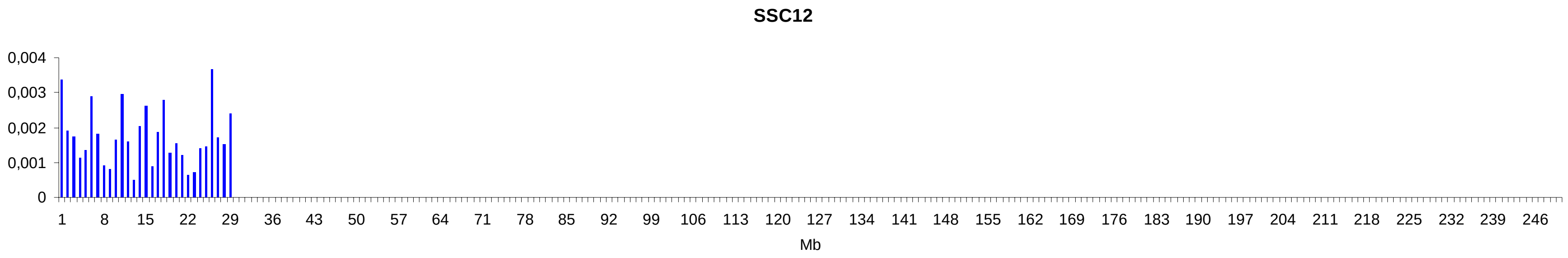
SSC12
0,004
0,003
0,002
0,001
0
1 8 15 22 29 36 43 50 57 64 71 78 85 92 99 106 113 120 127 134 141 148 155 162 169 176 183 190 197 204 211 218 225 232 239 246
Mb

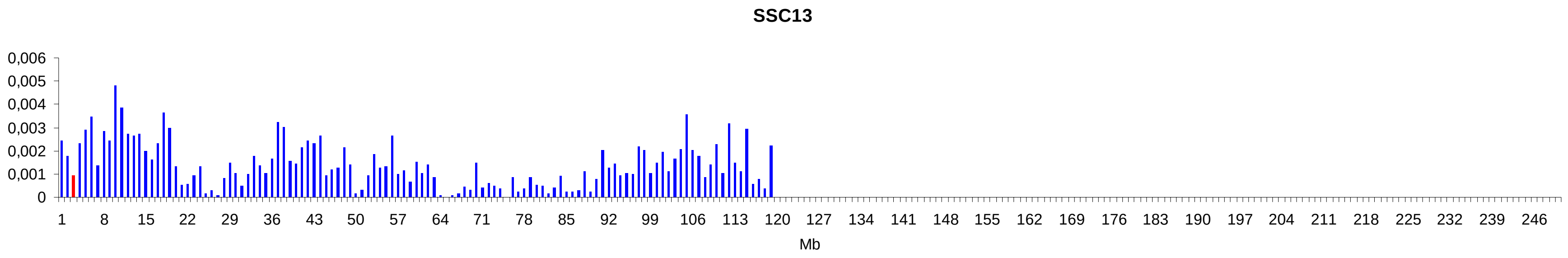
SSC13
0,006
0,005
0,004
0,003
0,002
0,001
0
1 8 15 22 29 36 43 50 57 64 71 78 85 92 99 106 113 120 127 134 141 148 155 162 169 176 183 190 197 204 211 218 225 232 239 246
Mb

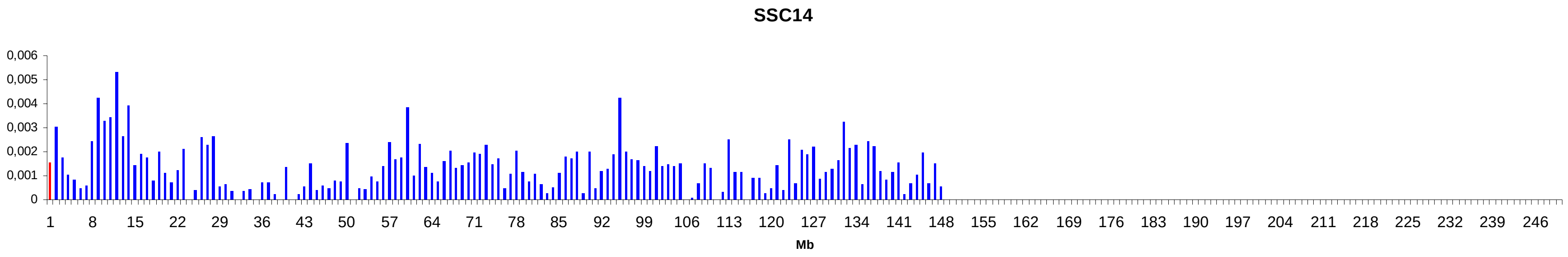
SSC14
0,006
0,005
0,004
0,003
0,002
0,001
0
1 8 15 22 29 36 43 50 57 64 71 78 85 92 99 106 113 120 127 134 141 148 155 162 169 176 183 190 197 204 211 218 225 232 239 246
Mb

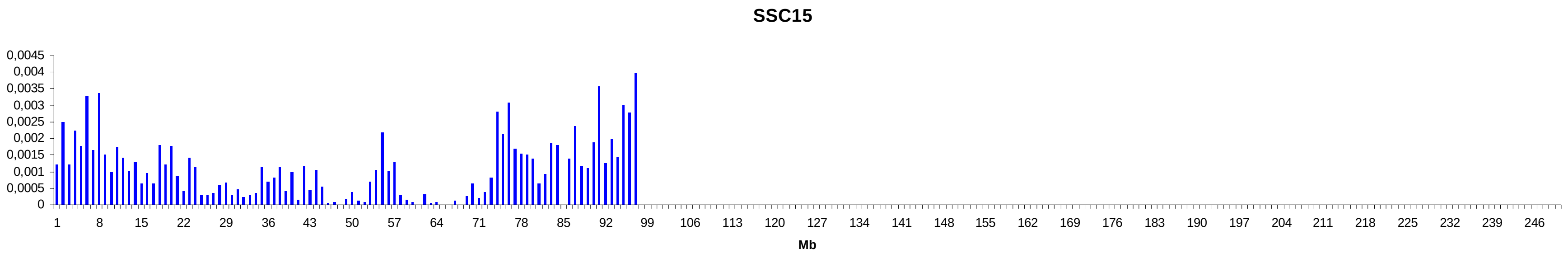
SSC15
Mb

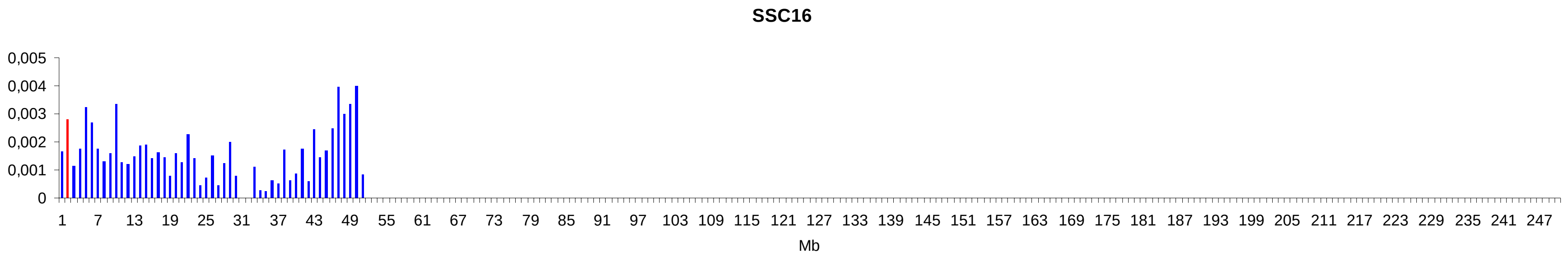
SSC16
Mb

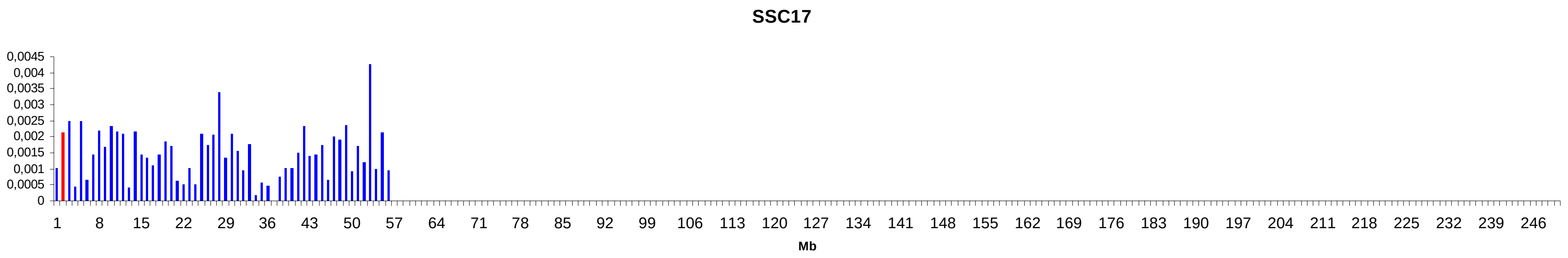
SSC17
Mb

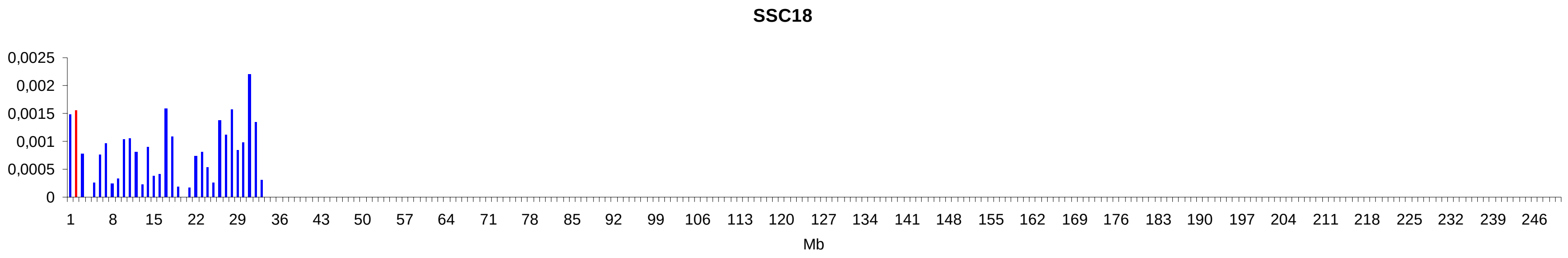
SSC18
0,0025
0,002
0,0015
0,001
0,0005
0
1 8 15 22 29 36 43 50 57 64 71 78 85 92 99 106 113 120 127 134 141 148 155 162 169 176 183 190 197 204 211 218 225 232 239 246
Mb

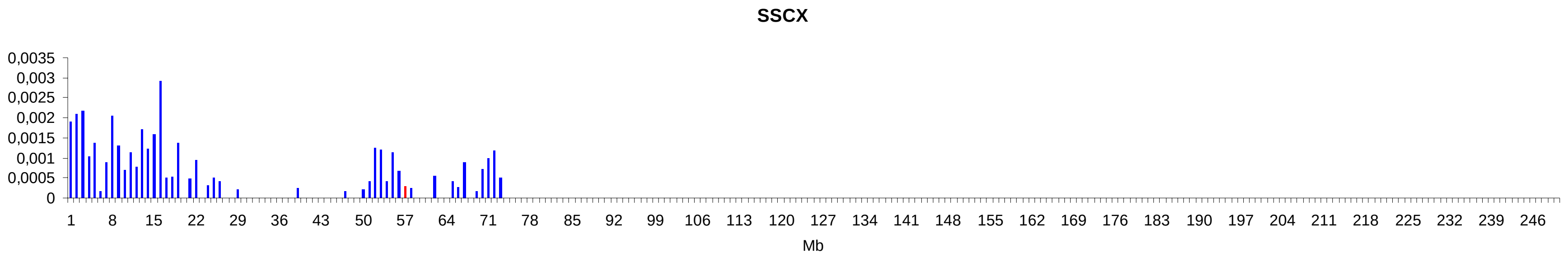
SSCX
0,0035
0,003
0,0025
0,002
0,0015
0,001
0,0005
0
1 8 15 22 29 36 43 50 57 64 71 78 85 92 99 106 113 120 127 134 141 148 155 162 169 176 183 190 197 204 211 218 225 232 239 246
Mb
